# Prevalence of Cardiovascular Disease Risk Factors in Migrants Participating in the PEP Family Heart Study, Nuremberg

**Published:** 2010

**Authors:** Gerda-Maria Haas, Klaus-Georg Parhofer, Peter Schwandt

**Affiliations:** 1 Arteriosklerose-Praeventions-Institute, Munich-Nuremberg, Germany; 2 Medical Clinic 2, University of Munich, Germany

**Keywords:** Prevention, Ethnicity, Risk factors, Cardiovascular diseases

## Abstract

**Objectives:**

The aim of this study was to assess the prevalence of cardiovascular risk factors in adults and their children from the 3 major groups of migrants participating in the PEP Family Heart Study^11^ and to compare the cardio-metabolic risk profiles between migrants and German participants.

**Methods:**

In this community-based cross-sectional study, anthropometric data, blood pressure and lipid profiles of migrants (480 children, 363 adults) from Turkey (TUR), Eastern Europe (EEU) and German immigrants from the former Soviet Union (GFSU) were compared with age and gender adjusted German (GER) residents (3253 children, 2491 adults).

**Results:**

The profile of risk factors differed considerably regarding specificity and frequency. The prevalence of ≥3 risk factors was as follows: in GFSU men 62%, women 36%, boys 19% and girls 17%; in TUR men 57%, women 30%, 15% boys and 6% girls; in GER men 48%, women 19%, boys 4% and girls 6%; for EEU men 38%, women 25% and 0% in children. No risk factor was present in GFSU men 13%, women 25%, boys 38% and girls 42%; TUR men 13%, women 28%, boys 27% and girls 22 %; GER men16%, women 45%, boys 46% and girls 41%; EEU men 17%, women 42 %, boys 29% and girls 27%. About 50% of the adults from Turkey and Eastern Europe were current smokers and one third of women and half of men from these two countries were overweight.

**Conclusions:**

The implementation of primary care measures for the prevention of cardiovascular disease in migrants is necessary, and it should consider the ethnic differences and the heterogeneous risk profiles.

## INTRODUCTION

Ethnic and regional variations in cardiovascular risk factors and disease substantially contribute to the different global burden of cardiovascular disease (CVD) in different populations.^1^ The European Case-Control Study demonstrated ethnic differences in risk factors for ischemic stroke.^2^ In different Asian populations the body mass index (BMI) cut-off for observed risk varies from 22 kg/m^2^to 25 kg/m^2^ and for high risk it varies from 26 kg/m^2^ to 31 kg/m^2^.^3^ South Asian descent present with a more adverse risk profile than those of European descent at the same BMI and/or waist circumference (WC).^4^ BMI cut points for obesity in terms of glucose and lipid profiles among 4 ethnic groups residing in Canada demonstrated a 6 Kg/m^2^ lower BMI cut point to define obesity among non-European groups compared with Europeans.^5^ Ethnicity-specific values for WC as measure of central obesity have been defined.^6^ Ethnic differences in triglyceride (TG) levels as reflection of insulin resistance are described, as well.^7^

Despite the knowledge that ethnicity matters in CVD morbidity and mortality among countries, most European cohort studies did not explore this connection further. There is, particularly in Europe, a shortage of information from cardiovascular cohort studies on racial/ethnic minority populations.^8^ Germany has become an important immigration country, the largest groups coming from Turkey (~2 million), the former Soviet Union (~3 million) and from other Eastern European countries.^9^ Among the 93 686 migrants living at the end of 2003 in Nuremberg, 22.8% had Turkish (TUR) origin, 19.3% came from Eastern Europe (EEU) except German migrants from the former Soviet Union (GFSU).^10^

The aim of this study was to assess the prevalence of cardiovascular risk factors in adults and their children from the 3 major groups of migrants participating in the PEP Family Heart Study^11^ and to compare the cardio-metabolic risk profiles between migrants and German participants.

## METHODS

The Prevention Education Program (PEP) Family Heart Study is a community- based cohort study designed to assess and improve cardiovascular health in children and their families. PEP enrolled first graders from 92% of the elementary school districts with a documented socioeconomic structure in Nuremberg. First graders, their siblings and parents were contacted through parent evenings in the schools at the beginning of each school year from 1994 to 2003. PEP was approved by the ethical committee of the Medical Faculty of the University Munich, the Bavarian Ministry of Science and Education, and the local school authorities. Written informed consent was obtained from all families participating in PEP.^11^ The study corresponds to standards of migrant sensitive research.^12^ Here we compare the cross-sectional data of 7087 participants (1416 men, 1938 women, 1863 boys and 1870 girls) who identified themselves as German (GER), Turk (TUR), Eastern Europeans (EEU) from Czech Republic, Slovakia, Poland, Hungary, Croatia, Bosnia, Slovenia or as Germans emigrating from the former Soviet Union (GFSU). Exclusion criteria were all other ethnicities, mixed ethnicity, overt malignant, cardiovascular, metabolic or endocrine diseases and incomplete data sets.

During the first visit at home age, gender, ethnicity, family medical history, personal medical history, educational status, professional status, socioeconomic status, physical leisure time activity and sedentary time, current medication and smoking status, including current smoking, passive smoking (living in a household with ≥ current smoker) and non- smoking were obtained using standardized questionnaires. All assessments were performed along the guidelines of the study manual by continuously trained research assistants.

Weight (scale SECA, Hamburg, Germany) and height (Stadiometer Holtain Ltd. UK) were measured in duplicate to the nearest 0.1 cm and 0.1 kg, respectively, with participants being barefoot and wearing light clothing. BMI was calculated as weight-to-height ratio (kg/m^2^). WC was measured to the nearest 0.1 cm according to the World Health Organization (WHO) recommendations at the end of expiration with a flexible inelastic tape (Fa. Siber Hegner, Switzerland) placed directly on the skin horizontal to the floor at the midpoint between the lowest rib and the iliac crest.^13^ Hip circumference (HC) was measured as the widest circumference over the major trochanters with the subject standing erect with abdomen relaxed and balanced on both feet with the feet touching each other and both arms hanging freely. Skin fold thickness (SFT) was measured on the left side of the body to the nearest 0.1 mm at three sites (triceps, biceps, subscapular) using a Lange caliper (Cambridge Sci. Industries, Maryland, USA). The ratio of subscapular and triceps SFT was taken as an index of truncal fat. Resting blood pressure (BP) was measured twice with appropriate cuffsizes according to arm size on both arms after 5 minutes rest recording the mean of the readings.

Venous blood was taken after an overnight fast within a strict time schedule prefixed according to the family’s preferred time in central school buildings on 6 Saturdays in November, December and January between 7:30 and 11:00 am. The early Saturday morning time was accepted by the participants, because of the fasting state which was repeatedly addressed as highly important. Venous blood was collected in different cooled tubes in cooling boxes (3°-4°C) and transported to the municipal sanitary board in Nuremberg for immediate centrifugation to obtain serum and plasma. Aliquots were transported on dry ice to the research laboratory in the Medical Clinic 2 of the University of Munich for storing either at -80° Celsius for later measurements respectively at 4° Celsius for lipid measurements within the following 3-4 days. Samples with a creamy chylomicron layer on the top after storage at 4° Celsius for 24 hours were excluded. As previously described,^14^ total cholesterol (TC) and triglycerides (TG) were measured by enzymatic methods (auto analyzer Epos, Eppendorf, Hamburg, Germany respectively later Alcyon, Abbott, Wiesbaden, Germany), HDL-cholesterol (HDL-C) after precipitation of apolipoprotein B containing lipoproteins by magnesium chloride and phosphotungstic acid. LDL-cholesterol (LDL-C) was calculated according to the Friedewald equation, if the TG concentration was <400 mg/dL(4.6 mmol/l), non HDL-C was calculated.

We have previously reported the definitions we used for adults and children,^14^ and here we provide it in brief. For adults risk factors were defined according to the criteria of National Cholesterol Education Program-Adult Treatment Panel (NCEP-ATP III)^15^ as low HDL-C (women<50 mg/dL, men <40 mg/dL), high LDL-C (>130 mg/dL), high triglycerides (TG >150 mg/dL), hypertension as systolic blood pressure (SBP) ≥140 mm Hg and/or diastolic blood pressure (DBP) ≥ 90 mm Hg, and/or use of medication prescribed for hypertension, high waist circumference (WC women >102 cm, men >88 cm), overweight (25-29.9 kg/m^2^), obesity (≥30 kg/m^2^), high Non-HDL-C (>156 mg/dL) according to Liu et al.^16^ Because in children blood pressure, weight, height and lipids change during growth and maturation, gender-, age- and ethnicity-specific cut off values had to be considered. Hypertension was defined as SBP and /or DBP >95^th^ percentile,^17^ overweight as >90^th^- 95^th^ BMI percentile and obesity ≥ 95^th^ BMI percentile according to the International Obesity Task Force.^18^ Abnormally large WC was defined as ≥75^th^ percentile.^19^

### 

#### Statistical Analysis

Continuous data are expressed as means ± standard deviation (SD) and frequencies as percent. Mean values obtained in different ethnic groups are compared by analysis of variance (ANOVA) and post-hoc tests. Frequencies are compared by Chi square test. Statistical analyses were performed by SPSS 15.0 version for windows (SPSS Inc., Chicago, Illinois) with 2-sided p values < 0.05 to be statistically significant.

## RESULTS

In general, in all ethnicities, the mean values of variables studied were significantly higher in male than in female adults and in female than male children and adolescents (Table 1).

**Table 1:** Characteristics (Mean ± SD) of participants with different ethnicities: the PEP Family Heart Study

Men n=1416	GER 1275	TUR 60	EEU 42	GFSU 39
Age (y)	39.2±6.9†	35.±6.3*	35.7±5.7*	39.9±7.0
Height (cm)	179.6±6.7†	171.6±5.4*†	177.2±6.1*†	176.8±5.6*†
Weight (kg)	83.4±12.2†	76.5±9.5*†	81.6±11.4†	83.8±10.9†
BMI (kg/m^2^)	25.9±3.1†	26.0±3.1	25.9±2.8	26.8±3.1
Waist Circumference (cm)	92.0±9.9†	91.6±8.8†	89.7±13.8†	95.9±9.5†
Hip Circumference (cm)	102.2±6.7†	100.8± 5.5	101.5±5.7	102.6±6.1
Waist to hip ratio	0.91±0.06†	0.91±0.06†	0.89±0.05†	0.92±0.05†
Biceps skinfold thickness (mm)	6.6±4.1	5.8±2.8	6.0±3.3	6.2±2.8
Triceps skinfold thickness (mm)	12.6±5.7	11.0±5.3*	11.5 ±4.4	12.5±4.8
Subscapular skinfold thickness (mm)	16.2±6.6	16.9±5.9	15.4±4.9	16.9±6.0
Subscapular/Triceps ratio	1.40±0.55	1.67±0.62*	1.43±0.45	1.44±0.52
Systolic Blood Pressure (mmHg)	131.5±14.3†	127.2±15.9*†	129.3±14.2†	130.2±11.2†
Diastolic Blood Pressure (mmHg)	85.5±10.0†	81.7±9.5*†	84.5±11.9†	84.2±11.0†
Total Cholesterol (mg/dL)	207.7±38.6†	199.4±38.8†	199.3±35.0	208.2±36.5
Triglycerides (mg/dL)	118.9±91.8†	160.0±81.2*†	106.4±55.2†	120.4±64.3†
HDL-Cholesterol (mg/dL)	48.8±11.7	41.2±9.6*	47.9±10.8	45.3±8.0
LDL-Cholesterol (mg/dL)	135.2±34.6†	126.2±32.9*†	130.1±32.0†	138.8±32.1†
NonHDL-Cholesterol (mg/dL)	159.0± 39.5†	158.2± 40.7†	151.4±36.6†	162.9±38.0†
TC/HDL-Cholesterol ratio	4.49±1.15†	5.11±1.65*†	4.34±1.16†	4.75±1.31†
TG/HDL-Cholesterol ratio	2.80±3.70†	4.36±2.93*†	2.40±1.47†	2.88±1.91
LDL/HDL-Cholesterol ratio	2.93±1.03†	3.24±1.18*†	2.86±0.97†	3.18±1.03†
**Women n=1938**	GER 1716	TUR 87	EEU 71	GFSU 64
Age (y)	36.9±7.1	34.0±7.2*	36.2±8.7	39.1±10.3*
Weight kg	66.3±12.8	64.2±11.5	65.0±11.0	69.4±13.1
BMI kg/m^2^	24.0±4.5	25.7±4.8*	24.4±4.1	25.7±5.1*
Waist Circumference (cm)	78.3±10.9	80.2±10.3*	78.9±10.1	82.7±12.9*
Hip Circumference (cm)	100.0±9.9	101.2±9.6	100.3±8.8	102.5±9.8
Waist to hip ratio	0.76±0.16	0.79±0.05	0.79±0.06	0.80±0.07*
Biceps skinfold thickness (mm)	9.6±5.1†	9.8±4.6†	9.5±4.4†	10.9±6.4†
Triceps skinfold thickness (mm)	18.7±6.7†	19.7±6.7†	18.8±6.1	20.5±7.3*†
Subscapular skinfold thickness (mm)	15.8±7.6	17.3± 7.6*	17.2±8.1	17.1±8.1
Subscapular/Triceps ratio	0.87±0.47	0.90±0.29	0.90±0.27	0.84±0.29
Systolic Blood Pressure (mmHg)	120.0±14.2	115.5 ±13.3*	123.8±20.3	120.6±16.1
Diastolic Blood Pressure (mmHg)	78.0±9.9	75.3 ±9.6*	79.6±11.2	78.0±11.1
Total Cholesterol (mg/dL)	194.3±34.2	183.0 ±30.1*	194.5±33.2	197.6±36.6
Triglcerides (mg/dL)	78.4±40.5	101.2±50.5*	83.1±37.1	84.0±34.7*
HDL-Cholesterol (mg/dL)	63.4±14.9†	52.4±14.3*†	61.2±14.0 *†	58.2±12.4*†
LDL-Cholesterol (mg/dL)	115.5±30.9	110.3±27.4	117.0±29.4	122.6±34.6
NonHDL-Cholesterol (mg/dL)	130.9±34.1	130.6±32.0	133.3±31.6	139.4±37.5*
TC/HDL-Cholesterol ratio	3.21±0.91	5.11±1.85*	3.29±0.78	3.55±1.05*
TG/HDL-Cholesterol ratio	1.36±1.00	2.17±1.48	1.44±1.01	2.93±1.58†
LDL/HDL-C ratio	1.95±0.77	2.29±0.98*	2.00±0.65	2.23±0.91*
**Boys n=1863**	GER 1621 (45%)	TUR 152 (22%)	EEU 45 (47%)	GFSU 45 (36%)
Age (y)	6.1±2.1	6.5±2.2*	6.1±1.8	6.5±2.2
Height (cm)	123.6±11.9	124.2±12.6	123.7±9.5	126.8±12.8*
Weight (kg)	24.9±7.0	27.3±9.7*	25.4±7.3	27.6±9.9*
BMI (kg/m^2^)	16.0±2.1	17.2±2.8*	16.3±2.1	16.8±3.2*
Waist circumference (cm)	57.2±6.2	60.0±9.1*	58.7±7.1	59.9±8.8*
Hip circumference (cm)	64.9±7.4	69.9±26.4*	66.0±7.1	67.8±9.30*
Waist to hip ratio	0.88±0.06	0.88±0.08	0.89±0.05	088±0.05
Biceps skin fold thickness (mm)	5.0±2.2	5.8±3.3*	5.3±2.1	5.8±2.8*
Triceps skin fold thickness (mm)	8.7±3.7	9.8±4.4*	9.1±4.4	10.0±4.9*
Subscapular skin fold thickness (mm)	5.9±3.1	7.1±3.7*	6.2±3.9	6.7±4.2*
Subscapular/Triceps ratio	0.72±0.28	0.75±0.20*	0.69±0.21	0.71±0.20
Systolic blood pressure (mm Hg)	103.8±9.2	105.2±9.9	105.8±9.5	106.9±10.0*
Diastolic blood pressure (mm Hg)	67.7±8.3	68.7±8.6*	68.5±8.4	67.5±8.6
Total Cholesterol (mg/dL)	174.3±29.3	165.9±23.4	177.0±31.6	169.1±26.1
Triglycerides (mg/dL)	61.5±28.8	72.9±34.6	61.1±29.6	64.6±23.3
HDL- Cholesterol (mg/dL)	59.5±14.0†	56.0±17.1	53.7±12.7*	57.2±8.8†
LDL- Cholesterol (mg/dL)	102.0±26.1	94.2±17.3*	111.1±28.7	98.9±26.8
Non-HDL-Cholesterol (mg/dL)	114.6±27.6	109.1±19.1	123.3±33.2	111.9±24.8
TC/HDL-Cholesterol ratio	2.92±3.35	3.20±1.02	3.47±1.03	3.00±0.48
TG/HDL-Cholesterol ratio	1.11±0.71	1.48±1.04*	1.26±0.89	1.17±0.46
LDL/HDL- Cholesterol ratio	1.83±0.75	1.89±0.84	2.21±0.88*	1.76±0.48
**Girls n=1870**	GER 1632 (47%)	TUR 161(22%)	EEU 45 (24%)	GFSU 32 (38%)
Age (y)	6.47±1.75	6.76±1.97	6.71±1.53	6.59±2.01
Height (cm)	122.7±12.1	123.3±12.9	123.5±11.1	123.8±14.2
Weight (kg)	24.5±7.2	27.0±9.8*	25.6±7.2	26.9±11.4
BMI (kg/m^2^)	15.95±2.24	17.22±3.29*	16.47±2.30	16.88±4.06
Waist circumference (cm)	56.2±6.1	59.4±8.5*	57.4±6.2	58.5±10.6*
Hip circumference (cm)	65.4±7.8	68.6±9.6*	66.3±7.6	67.5±11.3
Waist to hip ratio	0.86±0.06	0.87±0.05	0.87±0.07	0.87±0.06
Biceps skin fold thickness (mm)	6.0±3.3†	6.8±3.1*†	6.1±2.6†	6.1±3.9
Triceps skin fold thickness(mm)	10.3±3.8†	11.4±4.5†	10.3±4.5	10.1±5.5
Subscapular skin fold thickness(mm)	6.9±3.5†	8.6±4.9*†	7.2±4.0	7.9±6.0
Subscapular/Triceps ratio	0.7±0.3	0.8±0.2*	0.7±0.2	0.8±0.3*
Systolic blood pressure (mm Hg)	103.5±9.7	104.8±10.6	103.4±8.5	104.9±11.0
Diastolic blood pressure (mm Hg)	67.6±8.4	68.3±7.9	66.2±7.5	69.9±9.3
Total Cholesterol (mg/dL)	178.4±30.3†	166.8±31.5*	171.2±42.3	177.7±29.0
Triglycerides (mg/dL)	68.6±29.9†	76.9±35.0	70.6±30.01	67.0±15.8
HDL- Cholesterol (mg/dL)	56.7±14.4	53.7±12.2	47.5±12.7*	50.5±8.2*
LDL- Cholesterol (mg/dL)	107.8±27.2†	98.6±27.7*	109.6±36.4	113.7±27.1
Non-HDL-Cholesterol (mg/dL)	121.2±27.6†	113.9±30.2	123.8±40.4	127.2±28.3
TC/HDL-Cholesterol ratio	3.28±0.92†	3.24±0.89	3.83±1.20	3.57±069†
TG/HDL-Cholesterol ratio	1.27±0.69†	1.55±0.97	1.70±1.04*	1.39±0.50
LDL/HDL- Cholesterol ratio	2.06±0.87†	1.94±0.87	2.49±1.01	2.29±0.63†

GER: German, TUR:Turkish, EEU: Eastern Europe, GFSU:German origin emigrating from former Soviet Union

* P<0.05 for differences between ethnicities, within gender;

† P<0.05 for differences between gender, within ethnicity.

Prevalence of CVD risk factors among males and females from the four main ethnic groups of participants is shown in Table 2. In all ethnic groups, general adiposity (overweight plus obesity) was more frequent in men (56%-69%) than in women (30%-48 %) while central adiposity in terms of WC was two times higher in TUR and EEU women than in men. The prevalence of hypertension was 2-3 times higher in GER, TUR and GFSU men compared with women. Smoking was a common risk factor in both genders ranging between 30% and 60% in all groups. Dyslipidemia was frequent among adults including elevated Non-HDL-C in 38%-49% of men and 20%-33% of women.

**Table 2:** Prevalence of cardiovascular risk factors of participants with different ethnicities: the PEP Family Heart Study

Men	GER %	TUR %	EEU %	GFSU %
Overweight (BMI 25.0-29.9 kg/m^2^)	45.6	51.7	52.4	51.3
Obesity (BMI ≥ 30 kg/m^2^)	10.4	8.3	9.5	17.9
Waist circumference (>102 cm)	16.2	10.0	7.1	12.8
Hypertension (>140/90 mm Hg)	33.3	18.3	26.2	28.2
Active and passive* smoking	38.9	55.9	59.5	35.9
High LDL-Cholesterol (>130 mg/dL)	52.1	35.0	40.5	56.4
Low HDL-Cholesterol (<40 mg/dL)	21.2	51.7	23.8	28.2
High Non-HDL-C (>156 mg/dL)	49.4	48.3	38.1	48.7
Hypertriglyceridemia (>150 mg/dL)	21.8	46.7	16.7	28.2
**Women**				
Overweight (25.0-29.9 kg/m^2^)	20.7	27.6	32.4	28.1
Obesity (≥ 30 kg/m^2^)	9.4	19.5	9.9	20.3
Waist Circumference (>88 cm)	15.6	20.7	18.3	26.6
Hypertension (>140/90 mm Hg)	11.4	9.2	19.7	14.1
Active and passive* smoking	36.1	64.0	43.7	29.7
High LDL-Cholesterol (>130 mg/dL)	27.7	20.7	28.2	45.3
Low HDL-Cholesterol (<50 mg/dL)	17.6	49.4	12.7	31.3
High Non-HDL-C (>156 mg/dL)	20.0	21.8	22.5	32.8
Hypertriglyceridemia (>150 mg/dL)	4.8	14.9	2.8	9.4
**Boys**				
Overweight (BMI 90^th^ – 95^th^ perc.)	1.6	6.1	4.8	0.0
Obesity (≥95^th^ percentile)	3.1	12.1	9.5	25.0
Waist circumference (>75^th^ perc.)	16.0	12.1	33.3	43.8
Hypertension (>95^th^ percentile)	17.8	15.2	19.0	25.0
Passive smoking	26.0	51.5	19.0	6.3
High LDL (>130 mg/dL)	12.3	0.0	14.3	6.3
Low HDL-C (<40 mg/dL)	4.9	9.1	4.8	6.3
High Non HDL-C (<126 mg/dL)	29.9	12.9	42.9	31.3
Hypertriglyceridemia (> 150 mg/dL)	4.8	15.1	9.5	6.3
**Girls**				
Overweight (90^th^ – 95^th^ percentile)	1.7	5.6	18.2	0.0
Obesity (≥95^th^ percentile)	3.4	2.8	0.0	33.3
Waist circumference (>75^th^ perc.)	17.4	22.2	45.5	33.3
Hypertension (>95^th^ percentile)	24.7	19.4	13.0	33.3
Passive smoking	21.7	58.4	26.7	9.4
High LDL (>130 mg/dL)	16.5	11.1	18.2	33.3
Low HDL-C (<40 mg/dL)	8.6	16.7	27.3	16.7
High Non HDL-C (<126 mg/dL)	36.8	25.0	36.4	41.7
Hypertriglyceridemia (> 150 mg/dL)	8.4	16.7	9.1	0.0

GER: German, TUR:Turkish, EEU: Eastern Europe, GFSU:German origin emigrating from former Soviet Union

* ≥1 current smoker in the household

§see table 2 for percentage of children with complete lipid analyses in the 4 ethnicities

Among children from all ethnicities central adiposity in terms of WC ≥ 75^th^ percentile was more frequent than general adiposity. High blood pressure was remarkably frequent already in children and except GFSU children the prevalence of passive smoking was very high, especially in TUR children (more than 50%). Similar to adults, elevated Non-HDL-C was the most frequent form of dyslipidemia among children.

**Figure 1:**
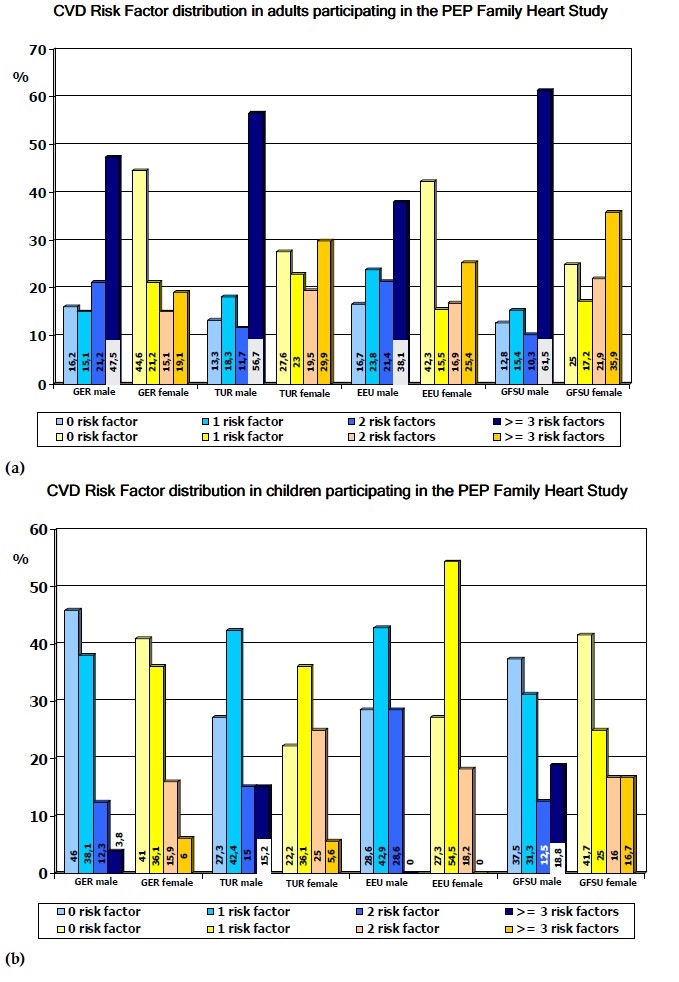
Frequency of cardiovascular risk factors in men (n=1531), and women (n=2138), boys (n=1663) and girls (n=1870) from German residents (GER) and migrants from Turkey (TUR) Eastern Europe (EEU) and German emigrants from the former Soviet Union (GFSU) participating in the PEP Family Heart Study

The risk profiles of adults and children are presented in Figure 1a and b. Men from all ethnicities had the worst risk profile in terms of ≥3 risk factors (between 38.1% and 61.5%), while in women the prevalence of ≥ 3 risk factors was substantially lower (between 19.1% and 35.9%). Children from all four ethnicities had a considerably better risk profile, though the majority of boys and girls had already 1 risk factor (between 31.3% and 42.9% in boys and 25.0% to 54.5% in girls). The comparison of the risk profiles among the 4 groups demonstrates that GFSU males had the worst risk profile (61.5% of men and 18.8% of boys had ≥3 risk factors). No risk factor was documented in 46% of GER boys, 44.6% of GER women, 42.3% of GFSU women and 41% of GER girls.

**Figure 2:**
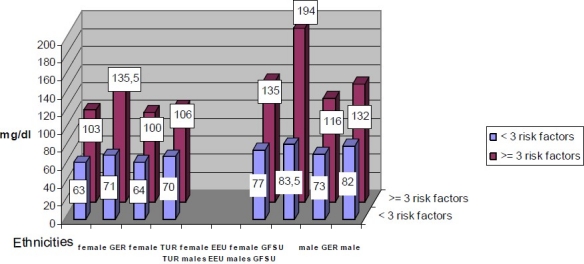
Triglycerides concentrations in men and women from 4 ethnic groups with < 3 risk factors, respectively ≥ 3 risk factors

Adults with ≥3 risk factors had higher TG concentrations in women (100-136 mg/dL) and men (116-194 mg/dL) compared with subjects with < 3 risk factors both in females (63-71 mg/dL) and males (73-84 mg/dL) in all ethnic groups (Figure 2). Furthermore TG concentrations were not substantially different between ethnicities according to WC tertiles. The prevalence of cardio-metabolic risk factors was highest in the top tertiles in men (GER 25.4%, TUR 20.0%, EEU 15.4% and GFSU 23.1%) and women (GER 11.9%, TUR 25%, EEU 17.4% and GFSU 41.2%) all ethnic groups. Among different ethnic groups, the top WC tertiles differed in men (GER >95 cm, TUR >94 cm, EEU >93 cm, GFSU >98 cm) and women (GER >80 cm, TUR >83 cm, EEU >84 cm and GFSU >88 cm).

## DISCUSSION

The PEP Family Heart Study^11^ is the first cross-sectional European study evaluating the prevalence of traditional cardiovascular risk factors in migrant parents and children from Turkey, Eastern Europe and German emigrants from the former Soviet Union in comparison to German resident families living in a German city with 500000 inhabitants. This study adds community-based data to improve the shortage of information from cardiovascular cohort studies on racial/ethnic minority populations in Europe.^8^ Between 1981 and 1994,Turkish migrants had a stable or decreasing CVD mortality which was lower than in the German population^20^ and a retrospective cohort study among GFSU migrants from 1990-2002 demonstrated that CVD mortality was lower than in the German population, but increased throughout the observation period and was higher for younger and lower for older GFSU migrants.^21^ However, still data on the risk factor profiles in these two cohorts are not reported.

The comparison of the characteristics of TUR, EEU and GFSU migrants with GER residents demonstrates that the anthropometric values were higher in GFSU and TUR women and boys, but similar in GER and EEU adults and children. BP was lower in TUR adults. Among lipids low HDL-C and high TG were most striking in TUR adults and children as well as in GFSU women who additionally had significantly higher LDL-C, non-HDL-C and LDL/HDL-C ratio and highest sum of skinfold thicknes. Lower HDL-C levels in women and children were the main difference between migrants from Eastern Europe and German residents.

The risk profile in GFSU migrants compared with GER residents was far more adverse: GFSU vs. GER men 62% vs. 48%, women 36% vs. 19%, boys 19% vs.4% and girls17% vs.6% had ≥3 risk factors. The prevalence of obesity was clearly higher in GFSU than in GER men (18% vs.10%), women (20% vs.9%), boys (25% vs. 3%) and girls (33% vs.3%) of similar age. However, prevalence of hypertension and of increased non-HDL-C is similar in men and children of both groups. The risk of hypertriglycridemia compared to normotriglyceridemic women having low HDL-C for GFSU was twice that of GER women, opposite to GFSU and GER men. Increased TG in GFSU adults had the strongest correlation with subscapular skinfold thickness supporting the adverse combination of between atherogenic hyperlipidemia (high TG and low HDL-C) and fat deposition among GER migrants from the former Soviet Union as compared to GER residents in Nuremberg. About one third of adults from both groups reported current smoking while passive smoking was by far more prevalent in GER children in agreement with two other studies in Germany.^9^ Because GER and GFSU adults participating in the PEP study are supposed to have the same ethnic origin, the more adverse risk pattern of the GFSU subjects also living for many years in Nuremberg might be due to an adverse risk factor transfer from their former habitat. This is opposite to the ‘healthy migrant hypothesis’ which is one of the supposed explanations for the formerly described lower mortality of migrants as compared to the resident population^20^ and supported by actual data from Nuremberg: migrants from GFSU and EEU contribute 16.5% of all deaths by myocardial infarction but represent only about 10% of the population.^21^ GFSU migrants had the highest prevalence of overweight and obesity beginning already in childhood.

Turkish adults participating in the PEP Family Health Study had higher plasma TG concentrations and lower TC, LDL-C and HDL-C levels than German participants. This corresponds to the lipoprotein profile of the adult Turkish populations,^22^,^23^ and is also in agreement with data from Turkish migrants living in Germany.^24^,^25^ Prevalence of hypertension in the PEP participants was lower in Turkish than in German men (18% vs.33%) and women (9% vs.11%) which is in agreement with Turkish migrants in Germany^24^ and which was recently confirmed by a direct comparison with Turkish adults.^26^ But the higher prevalence of low HDL-C (55.49% vs. 25.20%) in Turkish men/women vs. German men/women, the high rates of current smokers (56.64% vs. 39.6%) and 52% over-weight in men and 21% abdominal obesity in women are an adverse constellation for the Turkish migrants. This clustering of risk factors results in the worst risk profile among the four PEP-ethnicities: 61.5% of men and 35.9% of women had 3 or more CVD risk factors, and only 25% of men and 12.8% of women were without any risk factor. However, between 1981 and 1994 the CVD mortality rate of Turkish men declined by 18% and by 34% in West German men; the CVD mortality remained stable on a low level (of 45 per 100,000) in Turkish women compared to a 33% decline to 57 per 100,000 in German women.^19^ In Turkish adolescents aged 15 to 17 years the prevalence was 8% for hypertension, 15.8% for overweight and 3.4% for obesity.^27^

EEU migrants participating in the PEP study had a relatively favorable risk profile which is comparable with GER. Only about one third of the male and one quarter of the female participants had ≥ 3 risk factors; 42.3% of men and 16.7% of women were without any risk factor. This is very close to the risk profile of German men and women. Nevertheless 52% of men were overweight and 60% current active or passive smokers and about 30% of the 3 to 11 years old EEU children were passive smokers.

## CONCLUSIONS

The implementation of primary care measures for the prevention of cardiovascular disease in migrants is necessary, and it should consider the ethnic differences and the heterogeneous risk profiles. Future comparison of nutritional and physical activity habits between migrants and residents would be useful in explaining the environmental determinants of marked differences among various populations of migrants living in the same community. Longitudinal studies will clarify the importance of the ethnic differences documented even from childhood.
